# Evaluation of a Fatigue Detector Using Eye Closure-Associated Indicators Acquired from Truck Drivers in a Simulator Study

**DOI:** 10.3390/s21196449

**Published:** 2021-09-27

**Authors:** Łukasz Dziuda, Paulina Baran, Piotr Zieliński, Krzysztof Murawski, Mariusz Dziwosz, Mariusz Krej, Marcin Piotrowski, Roman Stablewski, Andrzej Wojdas, Włodzimierz Strus, Henryk Gasiul, Marcin Kosobudzki, Alicja Bortkiewicz

**Affiliations:** 1 Department of Psychophysiological Measurements and Human Factor Research, Military Institute of Aviation Medicine, Krasińskiego 54/56, 01-755 Warsaw, Poland; pbaran@wiml.waw.pl (P.B.); mdziwosz@wiml.waw.pl (M.D.); mkrej@wiml.waw.pl (M.K.); 2 Department of Aviation Psychology, Military Institute of Aviation Medicine, Krasińskiego 54/56, 01-755 Warsaw, Poland; pzielins@wiml.waw.pl; 3 Institute of Teleinformatics and Cybersecurity, Faculty of Cybernetics, Military University of Technology, Kaliskiego 2, 00-908 Warsaw, Poland; krzysztof.murawski@wat.edu.pl; 4 Department of Simulator Studies and Aeromedical Training, Military Institute of Aviation Medicine, Krasińskiego 54/56, 01-755 Warsaw, Poland; mpiotrowski@wiml.waw.pl; 5 Clinic of Otolaryngology, Military Institute of Aviation Medicine, Krasińskiego 54/56, 01-755 Warsaw, Poland; rstablewski@wiml.waw.pl (R.S.); awojdas@wiml.waw.pl (A.W.); 6 Institute of Psychology, Cardinal Stefan Wyszynski University, Wóycickiego 1/3, 01-938 Warsaw, Poland; w.strus@uksw.edu.pl (W.S.); h.gasiul@uksw.edu.pl (H.G.); 7 Department of Occupational and Environmental Health Hazards, Nofer Institute of Occupational Medicine, św. Teresy od Dzieciątka Jezus 8, 91-348 Łódź, Poland; marcin.kosobudzki@imp.lodz.pl; 8 Nofer Collegium, Nofer Institute of Occupational Medicine, św. Teresy od Dzieciątka Jezus 8, 91-348 Łódź, Poland; alicja.bortkiewicz@imp.lodz.pl

**Keywords:** car drivers, digital cameras, drowsiness, eye closures, facial features, fatigue detectors, PERCLOS, truck simulators

## Abstract

This paper presents a camera-based prototype sensor for detecting fatigue and drowsiness in drivers, which are common causes of road accidents. The evaluation of the detector operation involved eight professional truck drivers, who drove the truck simulator twice—i.e., when they were rested and drowsy. The Fatigue Symptoms Scales (FSS) questionnaire was used to assess subjectively perceived levels of fatigue, whereas the percentage of eye closure time (PERCLOS), eye closure duration (ECD), and frequency of eye closure (FEC) were selected as eye closure-associated fatigue indicators, determined from the images of drivers’ faces captured by the sensor. Three alternative models for subjective fatigue were used to analyse the relationship between the raw score of the FSS questionnaire, and the eye closure-associated indicators were estimated. The results revealed that, in relation to the subjective assessment of fatigue, PERCLOS is a significant predictor of the changes observed in individual subjects during the performance of tasks, while ECD reflects the individual differences in subjective fatigue occurred both between drivers and in individual drivers between the ‘rested’ and ‘drowsy’ experimental conditions well. No relationship between the FEC index and the FSS state scale was found.

## 1. Introduction

Driver fatigue and drowsiness are common causes of road accidents. Among over 36,000 people killed in motor vehicle traffic crashes on US roadways during 2019, 1.9% of fatalities involved a drowsy driver [1]. Police reports from European countries showed that 1% to 3% of all traffic accidents were caused by fatigue or drowsiness while driving [2]. In China, the traffic accidents caused by fatigue driving accounted for as much as 20% of the total number of accidents [3].

For almost 30 years, the issue of detecting driver fatigue and drowsiness has been investigated in many ways. Many research groups have proposed different technical solutions to detect driver fatigue early and thus minimise the risk of road hazards. In general, these solutions can be grouped into three categories according to the fatigue detection methods, which are based on monitoring (1) vehicle driving parameters, (2) driver physiological parameters, or (3) driver facial features [3,4,5,6,7,8,9,10,11]. The first category includes sensors for monitoring steering wheel touch, the steering wheel angle, the travel path and the vehicle’s speed [12,13,14]. Failure to detect a hand on the steering wheel or the detection of steering wheel turns beyond the lane may indicate unusual driver behaviour due to fatigue. The sensors for measuring driving parameters are easily mounted on a vehicle and relatively cheap; however, the data they provide may be affected by road or weather conditions, and these sensors should therefore be used together with devices of other categories [3,15,16].

The second group of methods is represented by electroencephalography (EEG), electromyography (EMG), electrooculography (EOG), electrocardiography (ECG), heart rate (HR), heart rate variability (HRV), and respiratory rate (RR) [17,18,19,20,21,22,23,24,25,26,27]. Each change in the mental state causes reactions reflected by changes in the values of physiological signals and their parameters, the intensity of which is an individual feature. EEG monitors are the most sensitive and reliable tool for assessing mental condition, including detecting fatigue. Although data provided by physiological parameter recorders are generally highly objective, these devices are expensive and require contact with the driver’s skin using electrodes. This significantly limits their use for the common detection of fatigue and drowsiness in vehicles [3].

Camera video recorders with software for analysing facial features belong to the third category of fatigue detectors [28,29,30,31]. Facial features change as fatigue increases. The continuous analysis of face images acquired from a driver while driving allows for the detection of fatigue without contact and effectively. At the beginning of the 1990s, the PERCLOS indicator—i.e., the percentage of time that the eyes were 80% to 100% closed—was adopted to research fatigue in drivers [32,33,34,35,36,37,38,39,40,41]. Later, ranges of 70% to 100% [42] and 75% to 100% [43] of eye closure were considered in other studies. Various research groups simplified the definition of this indicator to the percentage of time that the driver’s eyes were entirely closed over a certain period, typically 30 s or 60 s [44,45,46,47,48]. Other indicators for detecting fatigue were also proposed, the most measurable of which were the eye closure duration (ECD) and frequency of eye closure (FEC) [43,49,50,51,52]. When determining eye closure-associated indicators, most researchers considered only eye closures that lasted no less than 150 ms to 250 ms to distinguish them from ordinary eye blinks [47,53,54,55,56,57,58,59,60,61]. Nevertheless, eye blinks are also considered in the latest literature on driver fatigue research [28,62,63,64], as is eye tracking [65,66]. Additional information is obtained by tracking the position of the driver’s head.

In recent years, the method based on monitoring a driver’s facial features has been particularly actively studied. The rapid development of digital cameras and facial recognition software has provided an opportunity for this. The high potential of facial feature-based methods is evidenced by the involvement of car companies in the research and development of commercially available systems [31,67,68].

In this paper, we describe the design, construction and evaluation of a prototype fatigue sensor, the operating principle of which is based on the automatised detection of eye closures in the images of the driver’s face provided by a digital camera with an infrared (IR) illuminator.

## 2. Materials and Methods

### 2.1. Fatigue Detector

**Figure 1:**
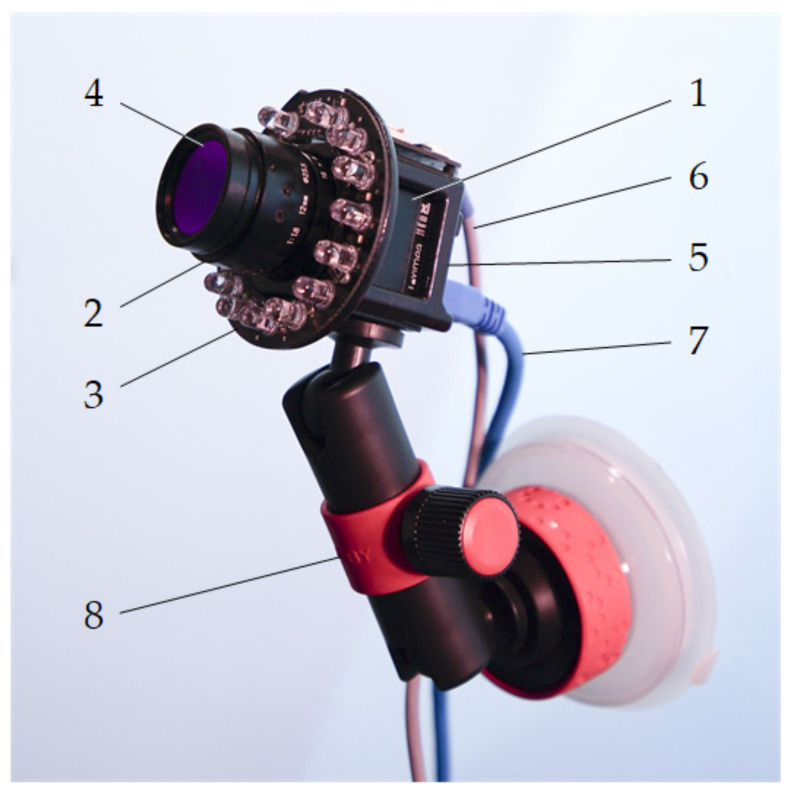
Main hardware components of the detector: 1—camera body; 2—lens; 3—IR illuminator; 4—band-pass filter; 5—supporting frame; 6—illuminator control cable; 7—camera data cable; 8—ball-joint holder.

The hardware part of the detector includes an MQ013RG-E2 camera by *Ximea* equipped with a 25 HB lens by *Tamron* and an IR face illuminator specially designed to be integrated onto the camera body. The main hardware components of the detector are shown in Figure 1. The camera body is cube-shaped with an edge length of 26 mm and weight of 26 g. The camera enables the capture of monochromatic images in visible-light and near infrared (NIR) frequencies with a resolution of 1280 × 1024 pixels at 60 frames per second (fps). The camera uses USB 3.1 communication to transmit the captured images to a personal computer (PC) and is powered by this link.

In order to improve the quality of images recorded in night conditions or in the darkened interior of the simulator cabin, we developed an IR illuminator with adjustable lighting intensity. The driver’s face was illuminated by 15 IR light-emitting diodes (LEDs) that were powered and controlled through an USB 2.0 port of the PC. The light reflected from the driver’s face was passed in the range of 820 to 910 nm through an F-BP850-25.5 filter by CCS Inc. Kyoto, Japan, mounted on the lens. The bandpass filter prevented the influence of ambient light. The illuminator was mounted on a supporting frame designed according to the Standard Template Library (STL) model and made using 3D printing technology. The supporting frame was mounted on a ball-joint holder with a suction cup to attach the detector to the front window of a car, truck or simulator. A more detailed technical description of the detector can be found in [69].

To operate the detector, we developed a software package that allowed us to record images, create calibration files, analyse the recorded data and visualise the results. To perform all the above-mentioned functions, the software package included the following parts:

- image acquisition module;
- calibration module;
- data analysis module;
- result visualisation module.

The software ran on the Microsoft .Net Framework 4.5.2 platform and was prepared in the C# language using the Visual Studio environment. The images captured by the sensor were saved on a PC disk in the form of video files in MP4 format using the image acquisition module.

The main task of the calibration module was to create calibration files—i.e., pairs of template images of the right and left eyes—which were later used to detect eye closures. The templates could be images of both the open and closed eyes of a given driver. Images of open eyes were more characteristic and hence we adopted them as the templates for detecting eye openings and counter states—i.e., eye closures. This approach was also supported by the fact that the determination of eye closure-associated indicators was based on the detection of both entire and partial eye closures. One or more pairs of the template images could be used to detect eye closures. More pairs increased the chance to detect eye closures effectively, but slowed down the identification procedure. Based on a series of trials, we decided to use three pairs of the template images to analyse each of the recordings. The templates were created from any three selected frames with clearly visible areas of open eyes. These areas were automatically selected by the commercially available FaceSDK 5.0.1 library by Luxand [70].

The same library was used in the data analysis module to detect the locations of the eyes in each frame of the video material recorded. Among the series of facial feature points, FaceSDK returned coordinates of the eye centres. In the case when the FaceSDK library could not find the facial feature points including the coordinates of the eye centres, such a frame could later be classified manually in the result visualisation module. Further analysis was performed using the template matching function covered in the OpenCV 3.3.0 library. This function operated in the mode of the normalised correlation coefficient calculation, and matched the areas around the centres of both eyes in consecutive frames of the video recording with the template images of the right and left eyes, respectively [71]. The result of the template matching function was a double-precision floating-point numeric value in the range from 0 to 1. The higher the value, the better the matching to the template image. In the OpenCV settings, a threshold value was defined at or above which the matching to the template image was considered sufficient and thus an eye opening was found. The images in each frame were sequentially matched to the three pairs of the template images in the loop until the template matching function returned a value equal to or greater than the threshold value. Then the returned value was adopted and no further matches were checked. In our study, the threshold value was set to 0.68. Hence, scores below 0.68 in all of the matching trials were qualified as eye closures. The template matching method was used for the right and left eyes separately. Finally, the analysis module treated the eyes as closed if both the eyes met this condition. More on the issues of classifying eyes as closed are discussed in the next section.

**Figure 2:**
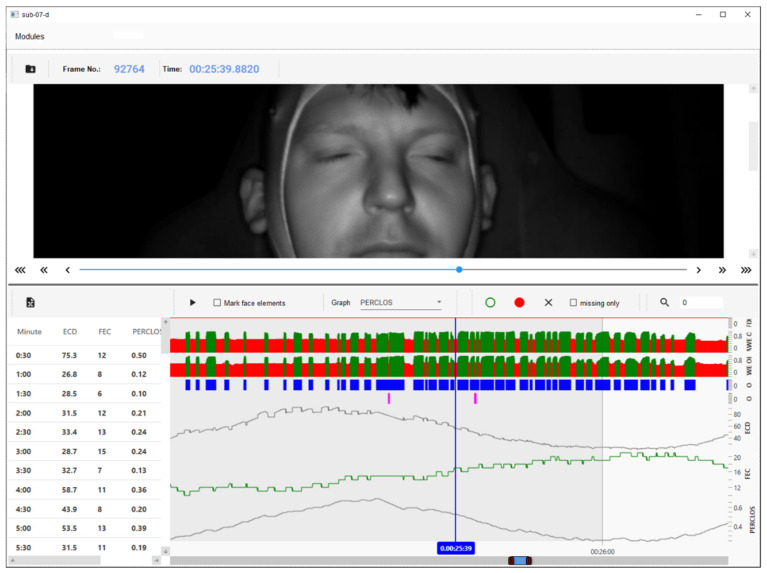
Window of the result visualisation module.

All unclassified frames as well as the automatically detected eye closures could be verified and corrected manually in the window of the result visualisation module, as shown in Figure 2. The upper part of the window displayed a frame of the recorded video material, selected with the cursor on any of the charts presented in the lower part of the window. In this area, automatically detected eye openings and closures were displayed as series of bars, the height of which reflected the degree of eye opening. Eyes classified as open were marked with green bars, while eyes classified as closed were marked with red bars. The previously unclassified eye states were unmarked, resulting in the gaps between the green or red bars. The distributions of these bars along with the successive video frames were presented for the right and left eyes, one below the other. The next chart in the form of a series of blue bars of the same heights indicated the video frames in which the driver’s eyes were open. The gaps between the blue bars indicated frames in which both eyes were qualified as closed. The purple bars below the blue ones indicated frames with ordinary eye blinks, which were not included in the determination of PERCLOS, ECD, and FEC indicators. The next three graphs showed the changes of these indicators along with successive video frames. On the left side of the charts, the average values of the eye closure-associated indicators, calculated within the time periods specified in the software settings, were listed. They could be exported to a comma-separated values (CSV) file.

### 2.2. Eye Closure-Associated Indicators

Below, we describe the indicators displayed by the result visualisation module and that are included in the data analysis.

PERCLOS indicates the percentage of time that the eyes are classified as closed within 30 s or 60 s time periods; we used 30 s time periods. When calculating PERCLOS, some researchers classify the eyes as closed if the eyelids cover the pupils by at least 70%, 75%, or 80%, with others doing so only if the pupils are entirely covered. The calculation of PERCLOS based on entirely covered pupils is, however, questionable, as fatigue or drowsiness makes the eyelids droop, and a tendency to partially cover the pupils by the eyelids is observed. On the other hand, it is often difficult to precisely determine the degree of pupil coverage in facial images due to the different orientation of the driver’s face relative to the camera that captures the images. The head position while driving, and thus the arrangement of the face elements—i.e., eyes, nose, mouth—in the captured images is an individual feature of each driver, which additionally changes while driving. Therefore, we use a threshold of 75% of the pupil coverage by the eyelids with a tolerance of approximately ±5% for the PERCLOS calculation. Moreover, we exclude eye blinks and eye closures that last 200 ms or less, i.e., 12 frames or less at 60 fps. Hence, PERCLOS is given by

PERCLOS = *N*_*closure*75,*duration*12_/*N*, (1)

where *N_closure_*_75,*duration*12_ is the number of frames in which the eyes are classified as closed with at least 75% coverage of the pupils and the duration of this closure is above 12 frames, and *N* is the total number of frames captured in a 30 s time period, i.e., *N* = 1800 at 60 fps. PERCLOS takes values from 0 to 1 or from 0 to 100 if it is expressed as a percentage. The first option is more common, and we use it when specifying PERCLOS values in this paper. PERCLOS is assumed to indicate fatigue when it takes values above 0.15 [49]. However, according to some reports, the indication of sleep deprivation and fatigue starts at 0.11 [41].

ECD is defined as the mean duration of clusters over 30 s or 60 s time periods, where a cluster is a set of continuous frames in which the eyes are classified as closed. As in the case of PERCLOS, when calculating ECD, we use 30 s time periods. The mathematical expression of ECD is also similar to PERCLOS, i.e.,

ECD = *N*_*closure*75,*duration*12_/*n*, (2)

except that *n* is the number of clusters in a 30 s period. In this approach, ECD is a number of frames, but can also be expressed as time in milliseconds. ECD is considered to be high when its value is well above 200 ms [49]. Some authors use 400 ms—i.e., 24 frames at 60 fps—as the fatigue recognition threshold. [51].

FEC specifies the number of eye closures recognised within 30-s or 60-s time periods, which are not ordinary eye blinks—i.e., that last longer than the predetermined value of 200 ms—corresponding to 12 frames. Within the 30 s period used to determine FEC, 2–3 eye closures are normal, while values above 5 may indicate fatigue in drivers [49].

### 2.3. Fatigue Symptoms Scales

The Fatigue Symptoms Scales (FSS) questionnaire was chosen to assess subjectively perceived levels of fatigue [72]. This questionnaire was constructed to measure different symptoms of subjectively experienced fatigue, both as a current state (FSS-S) and trait (FSS-T) [72]. Subjects are asked to indicate to what extent they experience a particular symptom on a five-point rating scale ranging from 0 (not at all) to 4 (very strongly). The questionnaire measures the overall level of fatigue experienced by the subject (total score), as well as individual and specific self-reported symptoms of fatigue included in a few subscales—e.g., cognitive, emotional, and physiological subscales. In the presented study, the raw score of the overall state scale of the FSS questionnaire was used for analysis as a subjective measure of the psychophysical state of the examined drivers.

### 2.4. Truck Simulator

**Figure 3:**
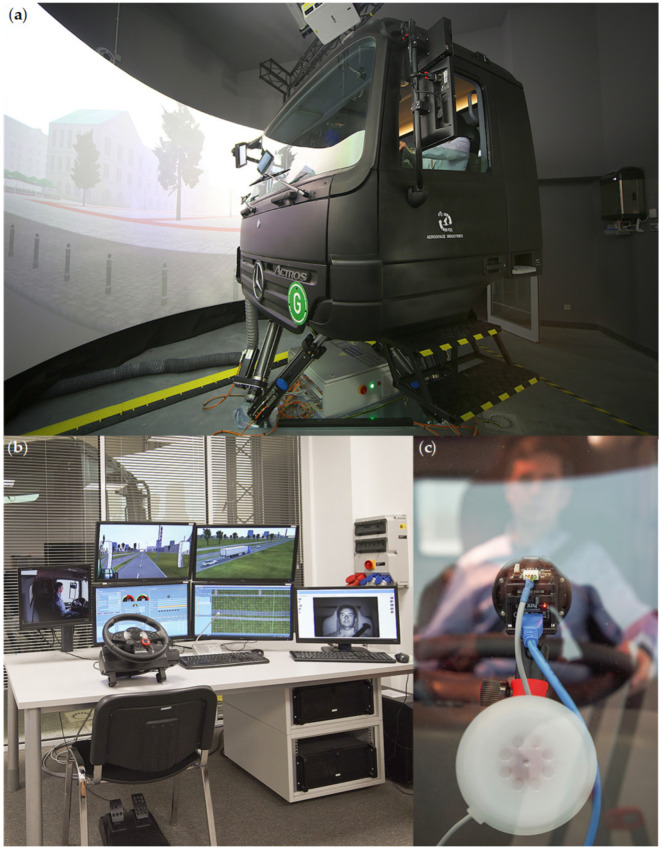
Photograph of the simulator system: the (**a**) exterior of the truck cabin, (**b**) instructor room, and (**c**) detector attached to the front window and directed towards the driver’s face.

The truck simulator used in this study is owned by the Nofer Institute of Occupational Medicine and was manufactured by ETC-PZL Aerospace Industries Sp. z o.o. The simulator is a stationary system that consists of a real Mercedes Benz Actros truck cabin placed on a movable platform with six degrees of freedom (6 DoF), advanced visualisation and acoustic background simulation modules, as well as an instructor room. The cabin with its cockpit and standard equipment has the same functionality as in a normal truck. The 6DoF platform allows the driver to feel rolls when turning, vibrations caused by uneven roads, or pitch of the cabin when braking. The images seen from the cockpit are projected by three laser projectors with widescreen ultra extended graphics array (WUXGA) resolution on a cylindrical screen with a horizontal field of view of 180°. The acoustic background simulation module generates sound effects heard by the driver and these sounds are reproductions of real sounds heard in the cabin, e.g., engine noise, sounds of other vehicles, and alarm signals of specific failures or malfunctions of the truck. The instructor or operator room is designed to control and operate the truck simulator. In this room, the PC that controls the fatigue detector was located. The simulator system is very similar to the one deployed at the Military Institute of Aviation Medicine, shown in Figure 3, which the authors described in the other paper [73].

### 2.5. Experimental Protocol

The experimental protocol complied with the Declaration of Helsinki and was approved by the Ethics Committee of the Military Institute of Aviation Medicine (Decision 11/2015). The study was carried out in the Nofer Institute of Occupational Medicine in accordance with the relevant guidelines and regulations. The subjects were informed in detail of the purpose and nature of the study and signed their informed consent for study participation and the use of identifying images in data analysis.

**Figure 4:**
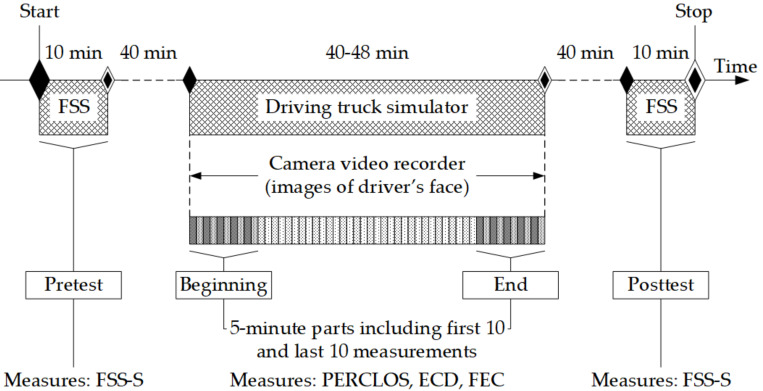
Scheme of the study. The procedure was repeated twice in states of rest (R) and drowsiness (D) at least a few days apart.

Eight professional truck drivers (men) aged 33.13 ± 4.39 (M ± SD) years were involved in the study. They were asked to drive the truck simulator twice: once when they were rested (R), and again when they were drowsy (D), i.e., after working a night shift. In the first iteration, the study involved five subjects who were rested and three who were drowsy, and the second iteration was the reverse, with five drowsy drivers and three rested ones. The study design is schematically illustrated in Figure 4. Approximately 40 min before the simulated truck driving, the subjects received the FSS questionnaire to complete the state scale, according to their own fatigue feelings, and then, they were prepared for driving. Before first main drive, the subjects were familiarised with the simulator and took a 5 min test drive. The subjects’ task during the main drive was to complete the route by following the trace that was being drawn in the upper middle part of the screen. The images of the drivers’ faces while driving were recorded with the detector attached by an operator to the front window inside the simulator cabin at the eye level of the drivers. The detector was wired to the PC located in the instructor room. From there, the operator controlled the image recording and also adjusted the intensity of the IR illuminator so that the face was clearly visible in the images.

The first part of the route (approximately 25% of the whole route) led along local roads through an urban area; the remaining part was along an expressway outside urban areas. The fastest subject completed the route in 40 min, and the slowest subject completed it in 48 min. However, the subjects were not required to complete the route as quickly as possible, but only to drive in accordance with the road traffic regulations. The subjects drove the same route when they were rested and drowsy, but with the requirement that the drives must be at least a few days apart. Approximately 40 min after the completion of the route, the subjects were again asked to complete the state scale of the FSS questionnaire. Relatively long intervals between the questionnaire and simulator phases of the study were employed to prepare the subjects for physiological recordings and remove all the measurement devices—e.g., an EEG electrode cap—as EEG signals were also acquired while driving. However, these recordings are beyond the scope of this paper.

As the subjective measurement of fatigue using the FSS-S scale was taken at two occasions, i.e., before and after completing the simulator task, the averages, i.e., arithmetical means of the first 10 measurements and of the last 10 measurements of PERCLOS, ECD, and FEC, were computed, and those averages were further treated as eye closure-associated indicators in the pre-test/post-test study design. Moreover, the categorical variable of the ‘rested’ or ‘drowsy’ states of the subjects was taken as the main factor describing the experimental conditions.

### 2.6. Statistical Analysis

Descriptive statistics methods were used for the evaluation of the PERCLOS, ECD, and FEC indicators, as well as the FSS-S scale, during the pre-test and post-test, considering the two experimental conditions. The Student’s *t*-test was used to assess the differences between the pairs of measurement occasions. Additionally, the validity of the FSS-S scale as a predictor of psychophysical state was checked with the receiver operator characteristic (ROC) curve analysis, with pre-test FSS-S scale result as a classifier and an experimental condition as an outcome variable.

The main goal of the detector evaluation was to check to what extent the eye closure-associated indicators could be used as predictors of the individual fatigue level and therefore the obtained data were analysed using a linear mixed model, which was chosen as an appropriate statistical method for repeated measurement schemes with time-varying covariates [74]. The model had three levels, with two measurement occasions—i.e., pre-test and post-test—nested in the ‘rested’ and ‘drowsy’ experimental sessions, and in the subjects. The ‘rested’ and ‘drowsy’ sessions were treated as a random factor. It was assumed that, for every subject, those two conditions could be seen as different points on the continuum of physiological fatigue. Raw scores of the FSS-S scale from pre-test and post-test were taken as the dependent variable, whereas the measurement occasion was treated as the time variable. Based on this model, three alternative models were estimated, with each one adding a different eye closure-associated indicator as an additional predictor. For those models, a change in the model-fit measures was computed, and their predictive properties were compared.

All analyses were performed using R statistical software version 4.0.3 by R Core Team. The ROC curve analysis was performed using the pROC package in version 1.16.2 [75]. The linear mixed model analysis was performed using the lme4 package in version 1.1-26 [76] and the lmerTest package in version 3.1-3 [77].

## 3. Results

We begin the presentation of the results by showing how PERCLOS, ECD, and FEC values changed during the simulator task. Table 1 graphically illustrates these changes for each of the subjects and for each of the experimental conditions—i.e., ‘rested’ and ‘drowsy’. Five-minute sections—i.e., the first 10 and the last 10 measurements—are marked in red. The average values of these parts of the PERCLOS, ECD, and FEC measurements were taken for the analysis. The trends of changes of the particular indicators increased in most cases, and they did not change only in some cases. A high variability of the indicator values was observed in some subjects. Particularly noteworthy are the cases of subjects no. 6 and 7 in the ‘drowsy’ state due to the exceptionally high values of the average PERCLOS achieved over the last five minutes of the simulator task. In subject no. 6, the average PERCLOS value over the first 5 min was 0.11, which followed an upward trend and reached 0.28 over the last 5 min. In subject no. 7, the average PERCLOS value over the first 5 min was 0.25, whereas over the last 5 min it was as high as 0.42. Thus, in subject no. 7, the starting value was already elevated, considering that PERCLOS above 0.15 indicated fatigue. The case of patient no. 7 in the ‘drowsy’ state also differed from the others due to the extremely high ECD values achieved at the beginning and at the end of the simulator task. A total 24 frames at 60 fps is the fatigue recognition threshold, whereas the average ECD values over the first 5 min and last 5 min were 41 and 72 frames, respectively. In addition, it should be noted that the case of subject no. 6 was distinguished by a high FEC value of 23, which increased by 12 from the beginning of the simulator task. FEC values above 5 are considered to show signs of fatigue.

The eye closure-associated indicators were firstly evaluated for mean and other distribution parameters as well as for the significance of differences between the measurements. The same analysis was then conducted for the FSS-S scale. The results are presented in Table 2. The effect size for paired samples—i.e., Cohen’s *d*—was computed using the approach suggested in [78].

A visible increase in the mean values of all the eye closure-associated indicators in both experimental conditions was observed. In the case of ECD in the ‘drowsy’ state, this increase was statistically insignificant due to the very high individual variability of the results. A significant increase from the beginning to the end of the experimental session in the ‘rested’ state was observed in the FSS-S scale. In the ‘drowsy’ subjects, the initial value of the FSS-S scale was very high in comparison to the ‘rested’ state and did not change significantly at the end of the experimental task.

When comparing the initial values in the ‘rested’ and ‘drowsy’ states, all the eye closure-associated indicators achieved higher values at the beginning of the experimental task in the ‘drowsy’ state. However, the differences were slight in terms of the effect size interpretation, and statistical significance was only found at the level of statistical tendency in the case of PERCLOS and ECD. Exact test values were: *t*_(7)_ = 2.078, *p* = 0.076, *d* = 0.520 for PERCLOS; *t*_(7)_ = 2.290, *p* = 0.056, *d* = 0.491 for ECD; and *t*_(7)_ = 2.555, *p* = 0.038, *d* = 0.476 for FEC. On the other hand, the subjective fatigue declared with the FSS-S scale was considerably higher at the beginning of the task in the ‘drowsy’ state—i.e., *t*_(7)_ = 4.800, *p* = 0.002, *d* = 1.055.

In the next step of the analysis, the ROC curve was computed to assess whether the distinction of the ‘rested’ and ‘drowsy’ states could be treated as valid—i.e., whether the subjective fatigue was concordant with the classification according to the study conditions. For this purpose, the preliminary (before the simulation task) score on the FSS-S scale was taken as a predictor, and the group classification (‘rested’/’drowsy’) was treated as an outcome variable. The ROC curve showed a significant predictive power for the FSS-S score, with an area under the curve (AUC) of 0.898 (bootstrap 95% confidence interval: 0.732–1.0). The optimal threshold was 7.5 points on the FSS-S scale with a specificity of 1.0 and sensitivity of 0.75. According to this result, it could be stated that the assignment to the experimental conditions was very well reflected in the subjective feeling of fatigue.

The main analysis was performed with the use of the linear mixed model, as described in the statistical analysis section. All consecutive models were estimated with the lmer function of the lme4 package in version 1.1-26, with the maximum likelihood estimation and default options for the optimisation method, which for lmer is a BOBYQUA (Bound Optimization BY Quadratic Approximation) algorithm delivered by the open-source NLopt library [79]. For the model evaluation, coefficients of determination in the form of the conditional and marginal *R*^2^ were used and computed using the equations given in [80]. Those coefficients were first proposed in 2013 in [81] as a simple and general way for presenting the explained variance both in linear and generalised linear mixed-effects models, with different link functions. The conditional *R*^2^ summarises the variance explained by an entire model, whereas the marginal *R*^2^ can be interpreted as the variance explained by fixed factors. Despite their simplicity and clarity, the coefficients do not provide information on the variance explained at each level of the linear mixed effects model. Therefore, according to the original suggestion presented in [81], they were supported by providing a simple proportion change of the variance—i.e., delta *R*^2^ calculated as shown for example in [82]—at different levels of analysed models.

At first, the zero mode—i.e., intercept-only model—was estimated, and intraclass correlations (ICCs) were computed. The ICC for the second level (task repetition) was 0.386 and the ICC for the third (person) level was 0.410, which showed that the similar amount of variation in the subjective level of fatigue was connected with individual differences, as well as with the study design and the repetition of experimental task under different circumstances.

Next, the model with the time variable was computed. This variable was tested for individual variability in time trends, but this random effect was found to be insignificant; thus, the time variable was included only as a fixed effect. The model with the time variable was not significantly better than the intercept-only model, with χ^2^_(1)_ = 2.024, *p* = 0.155. The conditional *R*^2^ of this model was 0.82, while the marginal *R*^2^—i.e., connected with fixed effects only—was 0.01. Thus, the considerable amount of variability in subjective fatigue was accounted for by the grouping structure and not by the changes during the course of the experimental task.

In the last step of the analysis, the PERCLOS indicator was added as a first-level predictor. This model showed an improvement on the previous model at the level of statistical tendency, with χ^2^_(1)_ = 3.573, *p* = 0.059. The conditional and marginal *R*^2^ were at the levels of 0.76 and 0.15, respectively. This showed that PERCLOS was visibly connected with the explaining variance of the FSS-S scores, as the increase in *R*^2^ for fixed effects was approximately 0.14. The variance decomposition showed that this improvement was mostly connected with the visible increase in the explained variance at the level of personal differences and at the level of experimental task conditions. The delta *R*^2^ was 0.37 at the second level—i.e., task conditions—and 0.33 for variance at the between-person level. The PERCLOS indicator did not further explain the residual variance of the model. The Akaike Information Criterion (AIC) for this model was 295.9.

Then, an alternative model with the ECD indicator instead of PERCLOS was estimated. This model was significantly better than the model with the time variable, with χ^2^_(1)_ = 5.673, *p* = 0.017. The conditional and marginal *R*^2^ were 0.77 and 0.20, respectively. The increase of the marginal *R*^2^ was approximately 0.19, making ECD a significant predictor connected with explaining the variability of subjective fatigue. The variance decomposition showed a visible increase in the explained variance at the level of personal differences (delta *R*^2^ = 0.63) and at the level of experimental task conditions (delta *R*^2^ = 0.22). The ECD indicator did not further explain the residual variance of the model. The AIC for this model was 293.77 and thus showed a slightly better fit than the model with PERCLOS as the main predictor.

Finally, the model with the FEC indicator instead of PERCLOS or ECD was estimated. This model did not show any improvement on the model with the time variable, with χ^2^_(1)_ = 0.883, *p* = 0.347. The conditional and marginal *R*^2^ were at the levels of 0.87 and 0.04, respectively. Hence, among the three eye closure-associated indicators, FEC had the lowest explanatory power in relation to the variance of subjective fatigue. The variance decomposition showed an increase in explained variance only at the residuals (within-person, within-conditions) level, with a delta *R*^2^ of 0.19, but the FEC indicator as a fixed-effect predictor was not statistically significant. The AIC for this model was 298.56, showing the worst fit among the alternative models and a poorer fit than the time variable-only model as a fixed-effect predictor.

**Figure 5:**
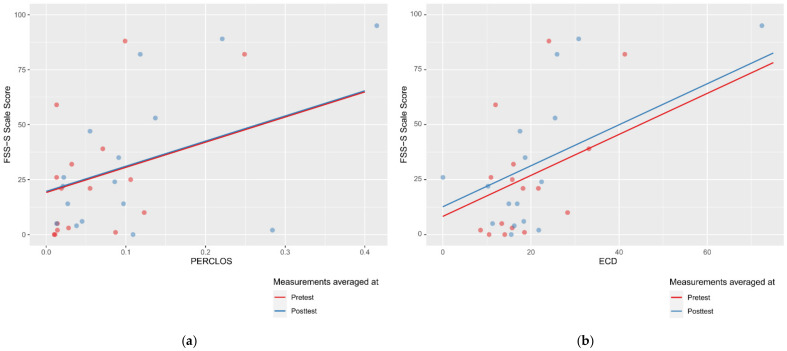
Estimated marginal effects for mixed linear models with (**a**) PERCLOS and (**b**) ECD as the main predictors.

The summary of all the analysed models is shown in Table 3, while the parameters of the final models with the PERCLOS, ECD, and FEC indicators are presented in Table 4, Table 5 and Table 6, respectively. The graphic representation of the models for PERCLOS and ECD is shown in Figure 5. The model for FEC was omitted due to the insignificant relationship between the main predictor and the response variable. It should be noted that an interaction between the time variable and one of the eye closure-associated indicators was tested for every model, but in any case, it did not improve the fit of the model; hence, it was omitted in the presentation of the results.

## 4. Discussion

### 4.1. Significance of the Results

We have presented the design and implementation of a driver’s face image recorder and tested its ability to detect fatigue based on eye closure-associated indicators. In our approach, we have checked to what extent these indicators could be used as predictors of individual fatigue levels. The results reveal that two of the three indicators used are closely related to fatigue, but they show different aspects of it. The PERCLOS indicator was proposed almost 30 years ago and has been well documented in the literature since then. Changes in PERCLOS acquired from the subjects at the beginning and at the end of the simulator task correlate with changes declared by them in the FSS questionnaire before and after the simulator task, respectively. In general, when driving a car, an increase of fatigue in drivers is observed, the intensity of which can be measured using the PERCLOS indicator. On the other hand, PERCLOS can also be used to determine the resistance of drivers to fatigue and indirectly to assess the difficulty level of the task performed.

In 2007, the authors of [50] observed that anomalous behaviours of drivers cannot be detected by using the PERCLOS indicator alone, except for the peaks over a threshold. They proposed the observation of two different indicators—i.e., ECD (introduced a year earlier in [49]) and FEC—in order to learn the model of normal behaviour of each driver and to give greater power to separate normal from anomalous behaviours. In 2014, the authors of [51] confirmed the high value of the ECD indicator in determining fatigue. The results of our study show that the ECD index well reflects the individual differences in subjective fatigue that occur both between drivers and in an individual driver between two experimental situations—i.e., in ‘rested’ and ‘drowsy’ conditions. Hence, ECD allows the differentiation of drivers in terms of fatigue and for a preliminary assessment to be made of the fatigue level at the beginning of a task. Depending on the needs, the ECD indicator can be used to analyse the quality of driving a car, as greater fatigue implies worse driving quality.

Subtle differences in sensitivity to various aspects of fatigue make PERCLOS and ECD an inseparable pair of indicators for detecting fatigue. Other authors, who do not exclude eye blinks from determining PERCLOS and ECD, point out the advantages of combining these two indicators for improving drowsiness detection [51]. The PERCLOS indicator is not strongly affected by eye state classification errors over a given period; that is, a few frames with eye state classification errors over the course of several hundreds of frames does not significantly influence the proportion of frames in which the driver’s eyes are closed for the period covered. However, in drivers who tend to blink frequently, the measured PERCLOS can be quite high, resulting in a false detection of drowsiness. On the other hand, ECD is not strongly affected by frequently blinking, but it can be significantly affected by eye state classification errors. When classification errors occur in the middle of closed eye sequences, the measured ECD can be significantly reduced, resulting in a failure to identify drowsiness. Our study from a different point of view confirmed the need to combine these two complementary indicators to improve the accuracy of fatigue and sleepiness detection.

In contrast, in our study it was not possible to prove the relationship between FEC and the state of fatigue of the subjects, although the authors of [50] have shown this indicator to be relevant for detecting fatigue. Due to the low diagnostic power of FEC revealed in our study, this indicator is not further considered.

### 4.2. Study Limitations

While it was possible to show a significant and valid relationship between subjective fatigue and eye closure-associated fatigue indicators, the low number of subjects and the high variability of the individual results make it hard to determine single critical values of PERCLOS or ECD indicators, which could serve as initial symptoms of increased fatigue. A larger, more representative sample that will provide normative data is needed to identify reliable warning thresholds for fatigue indicators, which in turn will allow the described sensor to be implemented to assess driver fatigue in real time and not only as a post-factum analysis.

Another limitation concerns the method of measuring fatigue. The declared current state of fatigue was assessed in a standardised manner before and after the simulator task, without controlling its state during the task itself. Such a scheme limits the possibilities of analysing changes in a driver’s fatigue levels to a linear trend only, and simultaneously constitutes the weakness of the questionnaire method itself. If the measurement of the subjectively perceived level of fatigue were carried out at certain time points during the whole simulator task, an ongoing fatigue state in response to various traffic situations as well as a more accurate link with eye closure-associated parameters could be gained. One possible method to obtain such measurements during a simulator task in a relatively non-invasive manner for the driver could be periodical requests to subjects to assess their psychophysiological condition on a several-point scale and, for experimenters themselves to simply record the responses. The continuous measurement of this variable in a car or truck simulator would enable observation of any increases or decreases in the driver fatigue level in relation to the duration of the study and the tasks performed in a virtual environment.

A hardware limitation of the detector is the need to adjust the height at which the camera is placed individually for the eye level of each subject. Nevertheless, this limitation will be overcome in the next version of the detector, e.g., by means of an optical devices optimised for the conditions of the truck cabin.

The above-mentioned limitations narrow the possibilities of analysis and interpretation, but the obtained results are sufficient to positively evaluate the fatigue detector and plan an extended study with the participation of a larger group of drivers.

## 5. Conclusions and Future Work

The camera-based sensor for continuous non-invasive fatigue detection allows drivers to monitor their condition in an automatised manner. Driver fatigue detection is realised by analysing values of the PRECLOS, ECD, and FEC indicators. The results show that the first two indicators have a diagnostic power, while the last one does not provide significant information on driver fatigue. Apart from confirming the effectiveness of the fatigue detector, one of the most important findings of the study is the complementarity of PERCLOS and ECD. Both PERCLOS and ECD could be treated as significant predictors of subjective fatigue reported by subjects while performing their assigned tasks. However, PERCLOS seems to be more sensitive to the changes in fatigue that can be observed in individual subjects during the course of the task, whereas ECD better captures between-person differences. Hence, these two indicators should be used together when assessing driver fatigue.

The novel character of the presented work relies on the synergy achieved by combining the in-house designed sensor with off-the-shelf solutions such as the FaceSDK library, as well as open-source codes represented by the template matching method. The proposed detector is distinguished by the readiness to integrate with any currently being developed system for assessing the psychophysical condition of the driver in simulated traffic conditions.

Plans for future work include extending the study to more than 30 drivers. The aim of the study with the participation a large group of drivers will be to determine the PERCLOS and ECD threshold values indicating fatigue while driving. The determination of the fatigue detection thresholds will be used by the constructors to equip the detector with new functionalities, allowing drivers to receive alerts when fatigue symptoms are detected, including orders to stop driving and rest. One of the main challenges will also be to develop a tool to measure subjective fatigue non-invasively while driving. Moreover, we plan to carry out experiments with simultaneous recording of facial images and acquisition of physiological data from drivers—i.e., HR, RR, galvanic skin response (GSR), and steering wheel grip force—to assess the level of mental workload caused by the driving itself.

## Figures and Tables

**Table 1:** Changes in the eye closure-associated indicators during the simulator task for each of the subjects in the ‘rested’ and ‘drowsy’ states.

Subject	State	PERCLOS (−)	ECD (Frame)	FEC (−)
1	R	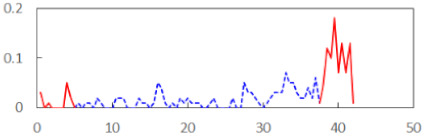	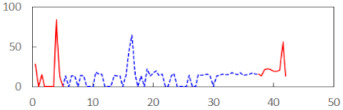	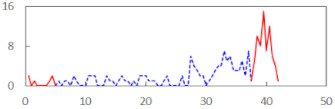
	D	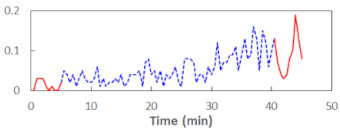	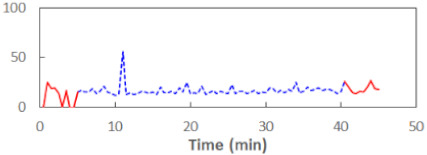	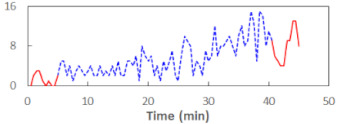
2	R	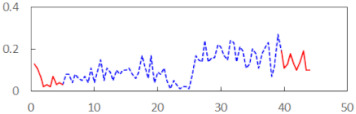	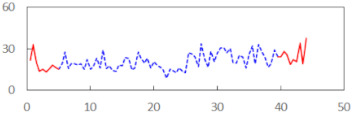	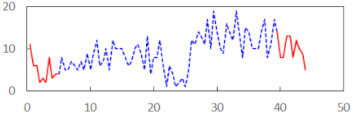
	D	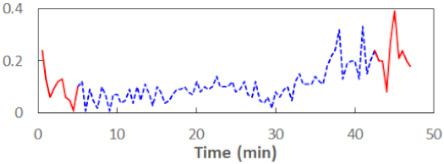	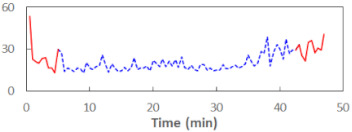	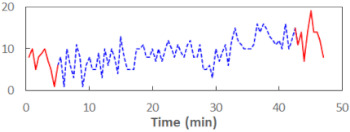
3	R	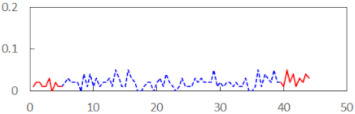	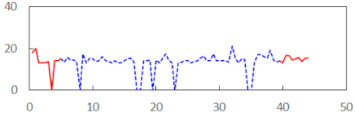	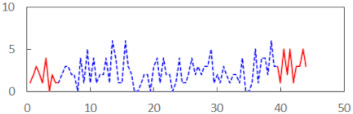
	D	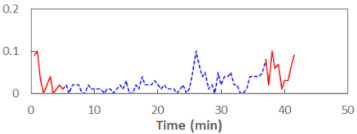	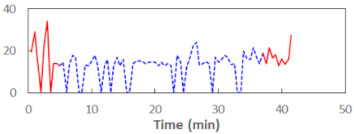	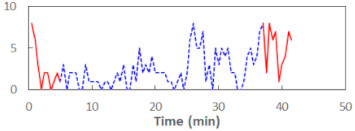
4	R	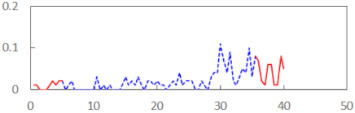	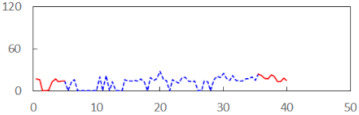	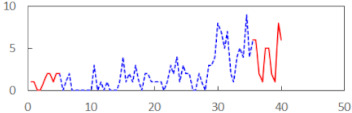
	D	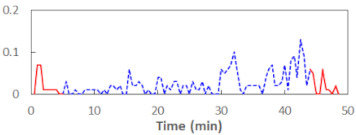	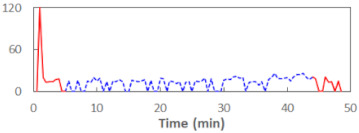	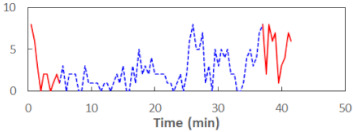
5	R	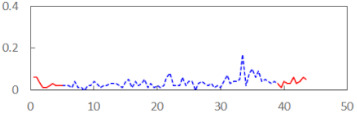	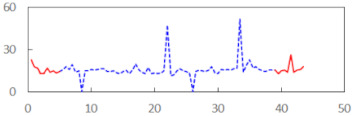	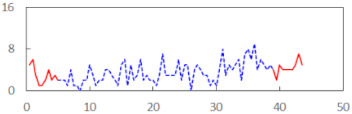
	D	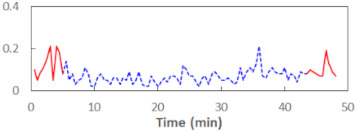	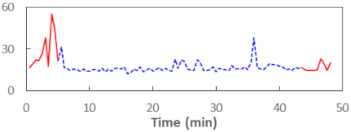	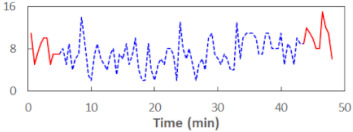
6	R	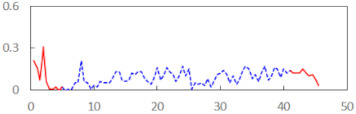	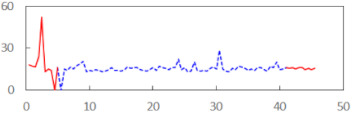	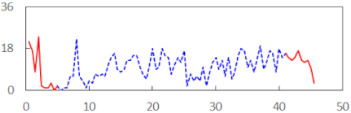
	D	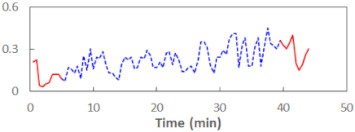	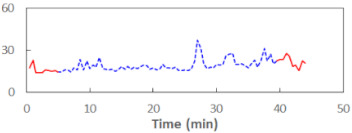	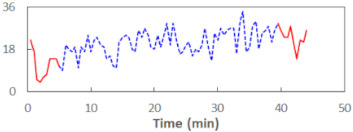
7	R	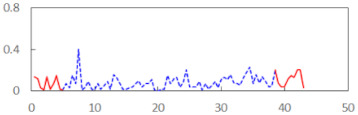	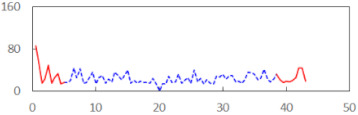	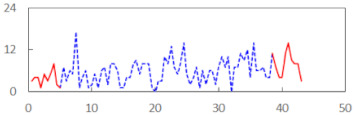
	D	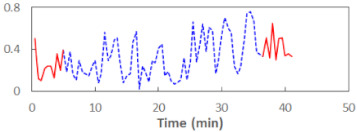	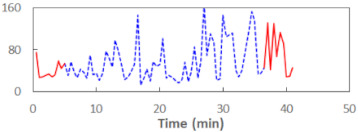	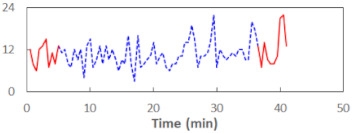
8	R	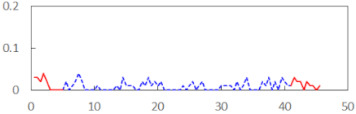	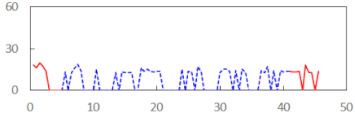	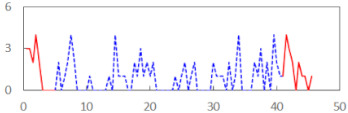
	D	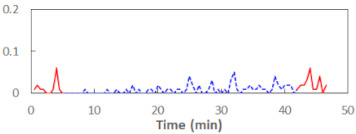	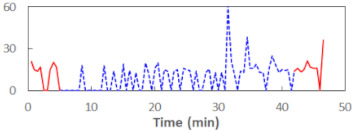	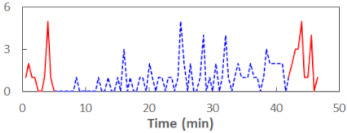

**Table 2:** Distribution parameters and significance of differences between measurements of the PERCLOS, ECD, and FEC indicators and the FSS-S scale under the established experimental conditions.

	Pre-Test		Post-Test				
					*t*	*p*	*d*
	Mean	SD	Mean	SD			
**Rested**							
PERCLOS	0.036	0.031	0.072	0.047	3.277	0.014	0.793
ECD	16.511	7.556	18.770	5.304	1.152	0.287	0.326
FEC	3.013	2.398	6.313	3.710	4.340	0.003	0.877
FSS-S	8.875	13.994	23.500	29.189	2.534	0.039	0.265
**Drowsy**							
PERCLOS	0.082	0.081	0.151	0.142	2.478	0.042	0.408
ECD	21.254	10.073	23.542	21.640	0.446	0.669	0.094
FEC	5.338	4.331	11.336	6.623	3.274	0.014	1.006
FSS-S	42.875	29.541	41.250	34.075	0.266	0.798	0.049

**Table 3:** Comparison of multilevel models for subjective fatigue.

Model	AIC	−2 Log Likelihood	df	χ^2^
M1 Intercepts only	297.5	289.46	4	
M2 Time variable	297.4	287.44	5	M2 − M1 = 2.024
M3 Final (PERCLOS)	295.9	283.90	6	M3 − M2 = 3.573
M4 Final (ECD)	293.8	281.77	6	M4 − M2 = 5.673 *
M5 Final (FEC)	298.6	286.56	6	M5 − M2 = 0.883

* *p* < 0.01.

**Table 4:** Results of the final three-level model for subjective fatigue with PERCLOS as a time-varying predictor.

Effect	Parameter Estimate	Standard Error	*t*-Value	*p* *(2-Sided)*	*95% Confidence Interval*	
					Lower	Upper
**Random effects at level 3 (subjects)**						
Intercepts	237.17	-	-	-	0.000	11,154.32
**Random effects at level 2 (task conditions)**						
Intercepts	216.98	-	-	-	2.503	899.07
**Random effects at level 1 (measurement occasions)**						
Residuals	177.71	-	-	-	91.85	404.97
**Fixed effects (averaged over task conditions and persons)**						
Intercepts	28.859	7.503	3.846	0.004	12.069	44.951
Time	0.532	5.443	0.098	0.923	−11.456	11.548
PERCLOS (scaled)	10.571	4.823	2.192	0.036	−0.410	−21.191

**Table 5:** Results of the final three-level model for subjective fatigue with ECD as a time-varying predictor.

Effect	Parameter Estimate	Standard Error	*t*-Value	*p* *(2-Sided)*	*95% Confidence Interval*	
					Lower	Upper
**Random effects at level 3 (subjects)**						
Intercepts	131.58	-	-	-	0.00	906.83
**Random effects at level 2 (task conditions)**						
Intercepts	269.56	-	-	-	41.08	931.88
**Random effects at level 1 (measurement occasions)**						
Residuals	163.98	-	-	-	84.66	380.96
**Fixed effects (averaged over task conditions and persons)**						
Intercepts	26.935	6.610	4.075	0.003	12.150	41.409
Time	4.380	4.591	0.954	0.356	−5.542	13.828
ECD (scaled)	11.611	4.183	2.776	0.009	2.089	21.631

**Table 6:** Results of the final three-level model for subjective fatigue with FEC as a time-varying predictor.

Effect	Parameter Estimate	Standard Error	*t*-Value	*p* *(2-Sided)*	*95% Confidence Interval*	
					Lower	Upper
**Random effects at level 3 (subjects)**						
Intercepts	347.27	-	-	-	0.00	1742.12
**Random effects at level 2 (task conditions)**						
Intercepts	474.89	-	-	-	99.49	1695.55
**Random effects at level 1 (measurement occasions)**						
Residuals	126.27	-	-	-	64.58	309.98
**Fixed effects (averaged over task conditions and persons)**						
Intercepts	23.374	9.225	1.534	0.031	2.713	43.387
Time	11.501	5.679	2.025	0.058	−3.261	23.299
FEC (scaled)	−5.697	4.622	−1.233	0.230	−15.892	7.399

## Data Availability

The data presented in this study are available on request from the corresponding author.
